# Supplementary material for: Phage Cocktails Constrain the Growth of Enterococcus
Source: mSystems. 2022 Jun 28;7(4):e00019-22. doi: 10.1128/msystems.00019-22 (PMC9426582; doi:10.1128/msystems.00019-22)
Supplement: TABLE S3 [file msystems.00019-22-st003.docx]

**Supplemental Table S3.**

| **Name** | **Family** | **Genus** | **Genome size** | **Accession No.** |
| --- | --- | --- | --- | --- |
| vB_OCPT_Bob | Myoviridae | Kochiodavirus | 150k | ON113169 |
| vB_OCPT_Car | Myoviridae | Kochiodavirus | 150k | ON113168 |
| vB_OCPT_Carl | Myoviridae | Kochiodavirus | 150k | ON113167 |
| EfV12-phi1 | Myoviridae | Schiekvirus | 150k | NC_048087.1 |
| vB_OCPT_Ben | Myoviridae | Schiekvirus | 150k | MN027503.1 |
| vB_OCPT_Bop | Myoviridae | Shiekvirus | 150k | ON125307 |
| vB_OCPT_Bill | Myoviridae | Shiekvirus | 150k | OM966901 |
| vB_OCPT_CCS1 | Myoviridae | Shiekvirus | 150k | ON113170 |
| vB_OCPT_SDS1 | Siphoviridae | Saphexavirus | 57k | ON113171 |
| vB_OCPT_SDS2 | Siphoviridae | Saphexavirus | 57k | ON113172 |
| vB_OCPT_CCS2 | Siphoviridae | Saphexavirus | 57k | ON113173 |
| vB_OCPT_CCS3 | Siphoviridae | Saphexavirus | 57k | ON113174 |
| vB_OCPT_Toy | Siphoviridae | Saphexavirus | 57k | ON113175 |
| vB_OCPT_CCS4 | Siphoviridae | Efquatrovirus | 40k | ON113176 |
| vB_OCPT_PG2 | Siphoviridae | Saphexavirus | 57k | ON113177 |
| vB_OCPT_PG9 | Siphoviridae | Saphexavirus | 57k | ON113178 |
| vB_OCPT_PG11 | Siphoviridae | Saphexavirus | 57k | ON113179 |
| vB_OCPT_PG13 | Siphoviridae | Saphexavirus | 58k | ON113180 |
| vB_OCPT_Ump | Podoviridae | unclassified | 18k | ON113181 |
