# Supplementary material for: Phage Cocktails Constrain the Growth of Enterococcus
Source: mSystems. 2022 Jun 28;7(4):e00019-22. doi: 10.1128/msystems.00019-22 (PMC9426582; doi:10.1128/msystems.00019-22)
Supplement: TABLE S2 [file msystems.00019-22-st002.docx]

**Supplemental Table S2**.

| **Strain** | **Species** | **Antibiotic Resistance** | **Genbank Accession** |
| --- | --- | --- | --- |
| EF09PII | *E. faecalis* | Tetracycline, vancomycin | n.a. |
| EF116PII | *E. faecalis* | Gentamicin-syn, tetracycline, vancomycin | n.a. |
| EF06PII | *E. faecium* | Ampicillin, penicillin G, tetracycline, vancomycin | n.a. |
| EF18PII | *E. faecium* | Ampicillin, penicillin G, tetracycline, vancomycin | n.a. |
| EF20PII | *E. faecium* | Ampicillin, penicillin G, tetracycline, vancomycin | n.a. |
| EF34PII | *E. faecium* | Ampicillin, penicillin G, tetracycline, vancomycin | n.a. |
| EF41PII | *E. faecium* | Ampicillin, gentamicin-syn, penicillin G, tetracycline, vancomycin | n.a. |
| EF48PII | *E. faecium* | Ampicillin, penicillin G, tetracycline, vancomycin | n.a. |
| EF50PII | *E. faecium* | Ampicillin, penicillin G, vancomycin | n.a. |
| EF79PII | *E. faecium* | Ampicillin, penicillin G, tetracycline, vancomycin | n.a. |
| EF98PII | *E. faecium* | Ampicillin, penicillin G, tetracycline, vancomycin | n.a. |
| B3286 | *E. faecalis* | Erythromycin, cefazolin, cefoxitin, clindamycin, gentamicin, gent synergy, oxacillin, trimethoprim/sulfa | GCA_000396365.1 |
| Ent6 | *E. faecalis* | Cefazolin, cefoxitin, clindamycin, gentamicin, oxacillin, trimethoprim/sulfa | n.a. |
| Tx1330 | *E. faecium* | Cefazolin, cefoxitin, clindamycin, gentamicin, oxacillin, trimethoprim/sulfa | GCA_003583905.1 |
| Yi6-1 | *E. faecalis* | Cefazolin, cefoxitin, clindamycin, gentamicin, gent synergy, oxacillin, tetracycline, trimethoprim/sulfa | GCA_000395095.1 |
| DP11 | *E. faecalis* | Gentamicin | JALPNV000000000 |
| DP6 | *E. faecalis* | -No resistant antibiotic found | n.a. |
| EF06 | *E. faecalis* | -No resistant antibiotic found | n.a. |
| EF11 | *E. faecalis* | Gentamicin | n.a. |
| TX2137 | *E. faecalis* | Cefazolin, cefoxitin, clindamycin, gentamicin, gent synergy, erythromycin, streptomycin synergy, tetracycline | GCA_000147595.1 |
| V587 | *E. faecalis* | Vancomycin, gentamicin, gent synergy, erythromycin, Cefazolin, cefoxitin, clindamycin, trimethoprim/sulfa | GCA_000394175.1 |
