# Supplementary material for: Phage Cocktails Constrain the Growth of Enterococcus
Source: mSystems. 2022 Jun 28;7(4):e00019-22. doi: 10.1128/msystems.00019-22 (PMC9426582; doi:10.1128/msystems.00019-22)

**Supplemental Table S1**.

**A)** Phage titers with host Yi6-1


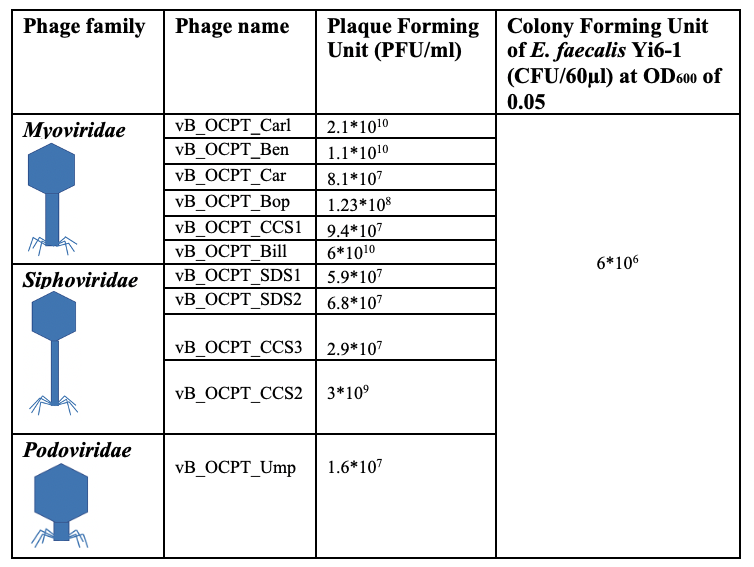


**B)** Phage titers with host EF06


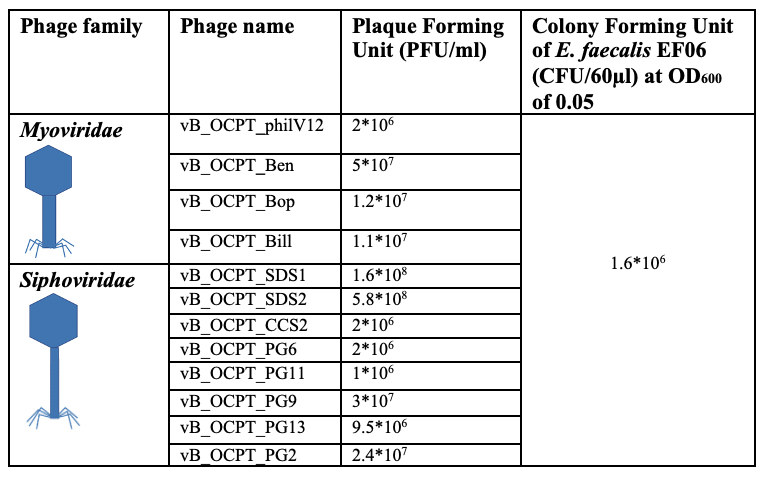


**C)** Phage titers with host EF11

**
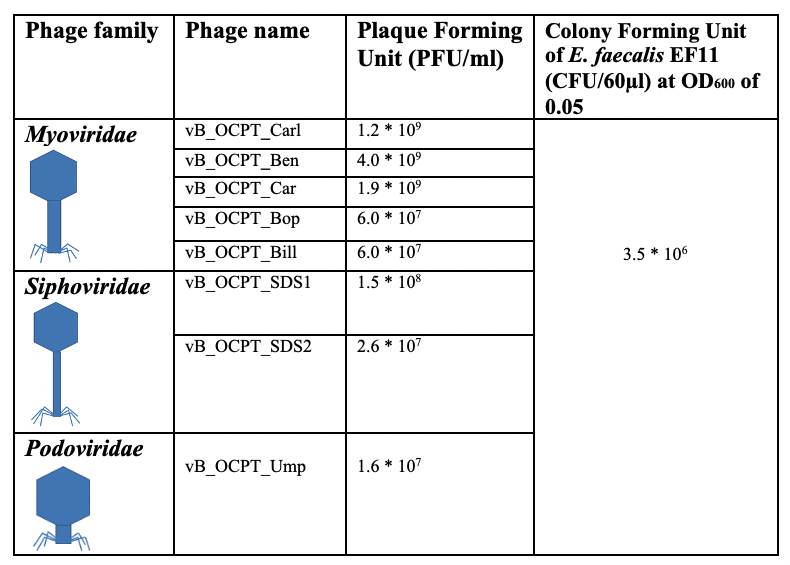
**

**D)** Phage titers with host V587


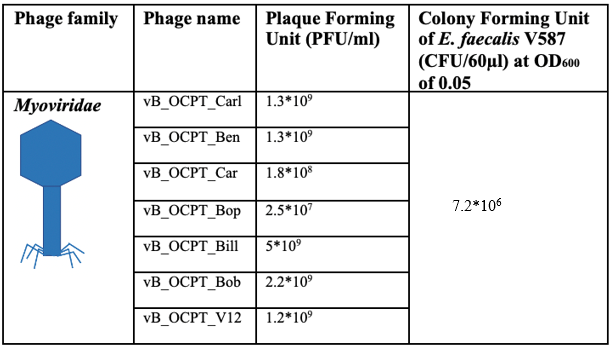

Supplement: TABLE S1 [file msystems.00019-22-st001.docx]
