# Supplementary material for: Phage Cocktails Constrain the Growth of Enterococcus
Source: mSystems. 2022 Jun 28;7(4):e00019-22. doi: 10.1128/msystems.00019-22 (PMC9426582; doi:10.1128/msystems.00019-22)
Supplement: TABLE S4 [file msystems.00019-22-st004.docx]

**Supplemental Table S4**. DNA primers (5’🡪3’) used to conduct qPCR experiments.

| **DNA primer** | **Sequence** | **Target** |
| --- | --- | --- |
| vB_OCPT_Ben_F | AAAACAGTTGAAACAGTTATT | Hypothetical protein |
| vB_OCPT_Ben_R | TATAAGCTTACTGTTTTACCA | Hypothetical protein |
| vB_OCPT_Bop_F | AACCGTTTGCTAACCATTTTC | hegA CDS |
| vB_OCPT_Bop_R | GGTTTGTGTACTAAGTGTATA | hegA CDS |
| vB_OCPT_Carl_F | CTAAAAATCTAACCAATTATC | Hypothetical protein |
| vB_OCPT_Carl_R | ATGGGTTTTTTCATTGCTAAA | Hypothetical protein |
| vB_OCPT_Bill_F | AAAACAGTTGAAACAGTTATT | Terminase |
| vB_OCPT_Bill_R | TATAAGCTTACTGTTTTACCA | Terminase |
| vB_OCPT_CCS1_F | TATGAGTAACATTAACATGGA | Lysin |
| vB_OCPT_CCS1_R | CCTGCCTTACGTAAAGAATCA | Lysin |
| vB_OCPT_SDS1_F | ACATTAACTCCTCTTTAGCTT | Hypothetical protein |
| vB_OCPT_SDS1_R | CGATTTTAGCATGCTGTTTCG | Hypothetical protein |
| vB_OCPT_CCS2_F | TTAGTAGGAAAGATTCTCTGT | Phage protein |
| vB_OCPT_CCS2_R | TAAAGGATTCAATTGACTTAG | Phage protein |
| vB_OCPT_Ump_F | TTAAAATGGAAGTGATTGTGG | Phage collar |
| vB_OCPT_Ump_R | TTAAAAAAGGCCACAGAGTTC | Phage collar |
